# Non-Volatile Memory Based on ZnO Thin-Film Transistor with Self-Assembled Au Nanocrystals

**DOI:** 10.3390/nano14080678

**Published:** 2024-04-14

**Authors:** Hui Xie, Hao Wu, Chang Liu

**Affiliations:** 1 Key Laboratory of Artificial Micro- and Nano-Structures of Ministry of Education and School of Physics and Technology, Wuhan University, Wuhan 430072, China; h.sieh@whu.edu.cn (H.X.); chang.liu@whu.edu.cn (C.L.); 2 Hubei Key Laboratory of Nuclear Solid Physics, Wuhan University, Wuhan 430072, China

**Keywords:** TFT memory, NVMs, nanocrystals, SoP, ALD

## Abstract

Non-volatile memory based on thin-film transistor is crucial for system-on-panel and flexible electronic systems. Achieving high-performance and reliable thin-film transistor (TFT) memory still remains challenging. Here, for the first time, we present a ZnO TFT memory utilizing self-assembled Au nanocrystals with a low thermal budget, exhibiting excellent memory performance, including a program/erase window of 9.8 V, 29% charge loss extrapolated to 10 years, and remarkable endurance characteristics. Moreover, the memory exhibits favorable on-state characteristics with mobility, subthreshold swing, and current on–off ratio of 17.6 cm^2^V^−1^s^−1^, 0.71 V/dec, and 10^7^, respectively. Our study shows that the fabricated TFT memory has great potential for practical applications.

## 1. Introduction

Recently, thin-film transistors (TFTs) based on transparent oxide semiconductors have been widely explored for pixel driving in display panels, showcasing significant promise for integration into system-on-panel (SoP) [1,2,3,4] owing to the inherent advantageous properties in oxide semiconductors, including high mobility, low process temperature, and transparency to visible light [5,6]. Among them, ZnO thin-film transistors have attracted extensive research interests due to their simple composition, non-toxicity, low cost, insensitivity to visible light, and the possibility of large-scale preparation [7,8]. ZnO is a wide and direct band gap semiconductor with a large excitation energy of ~60 meV, often crystallizes in the hexagonal wurtzite structure, and displays an intrinsic n-type conductivity [9]. A high degree of crystallinity is usually obtained in ZnO films, even deposited using relatively low temperatures [10]. Active-matrix liquid–crystal display (AMLCD) and active-matrix organic light-emitting diode (AMOLED) driving arrays based on ZnO TFTs have been successfully fabricated [11,12]. In a System-on-Panel, while TFTs can perform basic information processing to achieve comprehensive functionalities, devices capable of information storage are indispensable. Due to the similarity in structure to TFTs and ease of integration, TFT-based non-volatile memory offers great advantages for SoP [13,14,15,16,17,18,19].

The nanocrystals (NCs) charge-trap memory presents notable advantages over traditional floating-gate memory owning to discrete charge storage sites, particularly in terms of programming efficiency, endurance, and retention characteristics [20]. Various types of non-volatile nanocrystal memories have been explored, including semiconductor nanocrystals and metal nanocrystals. Metal nanocrystal memories, in particular, can offer significant benefits such as a wide range of available work functions, minimal energy perturbation due to carrier confinement, and enhanced coupling to the channel [21,22], which make them superior in charge storage ability. The dimension and shape of the metal NCs have a significant impact on the memory effect. The desired shape of NCs is typically spherical or quasi-spherical, which allows for uniform charge distribution and facilitates the trapping and retention of charge within the NCs. The spherical shape also minimizes defects and irregularities that could interfere with charge storage and retention, ensuring optimal memory performance [23]. When the size of metal NC is less than 4 nm, the Coulomb blockade effect will become pronounced, which raises the electrostatic potential of NCs, prevents the entry of additional electrons and influences program, erase, and retention characteristics [24]. Large-size NCs are favorable for large tunneling current and fast programming speed. The spacing of NCs should be larger than 4 nm so that lateral tunneling between NCs is negligible, and dense NCs are preferred to minimize the statistical variations. Thus, high density and large size are favorable when taking the trade-off with the NC number density into account [25]. Generally, the reported metal NC sizes ranged from 4 nm to 20 nm, and the density was in the order of 10^11^–10^12^ cm^−2^ [13,20,21,22,26,27,28]. 

To meet the needs of complex transparent and flexible electronic systems, non-volatile memories based on ZnO TFTs using metal nanocrystals as charge storage layers are beginning to attract attentions [26,27,28]. However, realizing high-performance ZnO TFT memory is still challenging. Park et al. presented a ZnO-based TFT memory using Al NCs with an average diameter of 7 nm and a density of about 1.6 × 10^12^ cm^−2^. A significant program-erase (P/E) window was achieved at low operating voltage [27]. Nevertheless, it suffered from poor retention and endurance characteristics due to the inferior quality of the tunneling oxide, worsened by damage incurred during the sputtering process of the ZnO channel. Al metal particles formed by sputtering are prone to oxidation in subsequent processes, leading to alterations in its characteristics. In addition, the low work function of Al significantly contributed to the observed decrease in retention time. Kang et al. investigated a ZnO TFT memory based on a top-gate structure [28]. Sputter-condensed Pd nanoclusters with an average size of 5 nm and density of 1.0 × 10^12^ cm^−2^ were utilized as the charge storage medium, and 5.8 V P/E window was obtained under +18 V/200 ms and −18 V, 200 ms P/E operations, indicated that more than 13 electrons were trapped in each Pd nanocluster. However, the deposition process of the tunneling oxide layer inevitably caused physical damage to the active channel, resulting in inadequate on-state characteristics, including channel current and subthreshold swing. 

For TFT memory to be practically applied in flexible and transparent electronic systems, both good non-volatility and sufficient on-state characteristics are required. Therefore, a charge storage medium with excellent charge storage ability and immunity to charge leakage is needed, along with optimal fabrication methods and process flows, to achieve good overall performance. Among various metal NCs, Au NCs exert a great memory effect owing to their stable chemical properties [29]. With a high density of states around the Fermi level, Au NCs possess an excellent capacity to store electrons [21]. Moreover, the high work function of Au NCs enables the formation of a deep quantum well between the tunneling and blocking oxide layers, ensuring minimal charge loss of the memory device during retention. Atomic layer deposition (ALD) is a chemical deposition technique based on layer-by-layer, self-limiting and saturated surface reactions, which can be used to grow dense, pinhole-free, and conformal thin films [30]. More importantly, ALD minimizes material damages during fabrication compared to methods like sputtering, which reduces the traps in the films. ZnO thin-film transistors fabricated by ALD presented superior transistor performance [8,31]. Furthermore, by employing high-reactivity precursors in ALD, high-quality oxide films can be grown at very low temperature [32]. Together with the capability of large-scale deposition, ALD is considered an optimal method for the fabrication of transparent flexible electronic devices [33].

Here, we present a non-volatile memory based on a ZnO thin-film transistor prepared at low temperature, for the first time, with self-assembled Au nanocrystals as the charge storage layer. Both the ZnO channel and the AlO_x_ oxide layer in the memory were prepared by ALD to ensure good thin-film quality. The results show that well-isolated Au nanocrystals were formed self-assembly by annealing at a low temperature of 250 °C, with an average size of 8 nm. The TFT memory shows excellent charge storage capability, including a P/E window of 9.8 V at a programming voltage of 15 V, a P/E window of 7 V extrapolated to 10 years, and a P/E window of 9.7 V after 10^4^ P/E cycles. In addition, the TFT memory presents favorable on-state characteristics with field-effect mobility of 17.6 cm^2^V^−1^s^−1^ and I_ON_/I_OFF_ exceeding 10^7^. The superior non-volatility and on-state performance make it promising for applications in transparent and flexible electronic systems.

## 2. Materials and Methods

### 2.1. Device Fabrication

**Figure 1:**
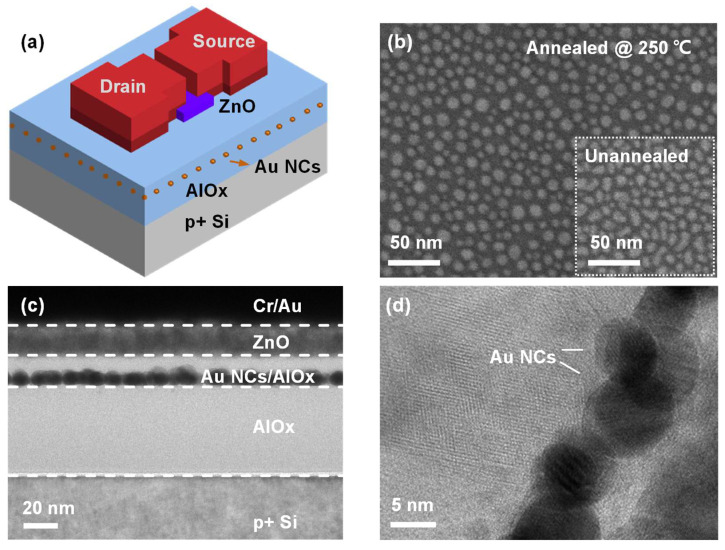
(**a**) Schematic of the proposed TFT memory; (**b**) SEM image of Au NCs on AlO_x_ blocking oxide after annealing, insert is the SEM image of the unannealed sample; (**c**) cross-sectional TEM image of the fabricated TFT memory; (**d**) high-resolution TEM image of Au NCs in the TFT memory.

The schematic structure of the proposed ZnO TFT memory is shown in Figure 1a. The device adopted a staggered TFT structure, with heavily doped p-type silicon (0.001 Ω∙cm–0.005 Ω∙cm) serving as the bottom gate. For the fabrication, firstly, 50 nm AlO_x_ was deposited on RCA-cleaned p+ silicon substrate by ALD (TFS200, Beneq, Espoo, Finland) at 200 °C, with Trimethylaluminum (TMA) and H_2_O as precursors, which served as the blocking oxide layer. A cycle of AlO_x_ deposition consisted of 0.7 s pulse of TMA, 10 s of purging using N_2_, 1 s pulse of H_2_O, and 15 s purging using N_2_. The growth rate of AlO_x_ was 0.12 nm per cycle, as determined by thickness measurement by an ellipsometer. Subsequently, for the fabrication of Au NCs, a 1.5 nm Au layer was deposited by thermal evaporation (JSD-400) at room temperature under a vacuum of 2 × 10^−4^ Pa and followed by rapid thermal annealing in an N_2_ atmosphere at 250 °C for 1 min. Next, the 10 nm AlO_x_ tunneling oxide layer was deposited by ALD at 250 °C, using the same precursors and deposition parameters. Next, 16 nm ZnO was deposited on the tunneling oxide by ALD at 100 °C in the same chamber without vacuum breaking, with Diethylzinc (DEZn) and H_2_O as precursors. The pulse times of DEZn and H_2_O were both 0.3 s, and the following N_2_ purging times were 20 s and 25 s, respectively. The measured growth rate of ZnO was 0.13 nm per cycle. The growth parameters of AlO_x_ and ZnO are summarized in Supplementary Table S1. More details about the process can be found in our previous paper [31].

After the deposition of all functional layers, the ZnO layer was patterned using photoresist (S1818, SHIPLEY, Tustin, CA, USA) and UV lithography(H93-37, Nanguang, China) and then wet etched (H_3_PO_4_, 10%) into 150 μm × 300 μm cubes, which served as the isolated active channel layer for the TFT memory. Then, source and drain electrode patterns were created by photoresist (N4340, Allresist, Strausberg, Germany) and UV lithography. Next, 10 nm Cr and 50 nm Au were prepared using electron beam evaporation on the patterns at room temperature, under a vacuum of 2 × 10^−4^ Pa, and the S/D electrodes were formed by a lift-off process. The channel length and width of the device were 50 μm and 200 μm, respectively, which were defined by the spacing and width of source and drain electrodes. The process flows are illustrated in Supplementary Figure S1. In addition, the control device without Au NCs was also fabricated simultaneously. No post-annealing was involved during the fabrication process of the devices. 

### 2.2. Device Characterization

The device structure was characterized by a field emission transmission electron microscope (JEM-F200, JEOL, Tokyo, Japan) with an acceleration voltage of 200 kV, the morphological characterization of metal nanocrystals was performed by scanning electron microscopy (Sirion, FEI, Hillsboro, OR, USA). The thickness of the AlO_x_ film and ZnO film were measured by an ellipsometer (Alpha-SE, J.A. Woollam, Lincoln, NE, USA), and the electrical characteristics of the memory device were measured with a semiconductor characterization system (Keithley 4200scs, Cleveland, OH, USA).

## 3. Results and Discussion

**Figure 2:**
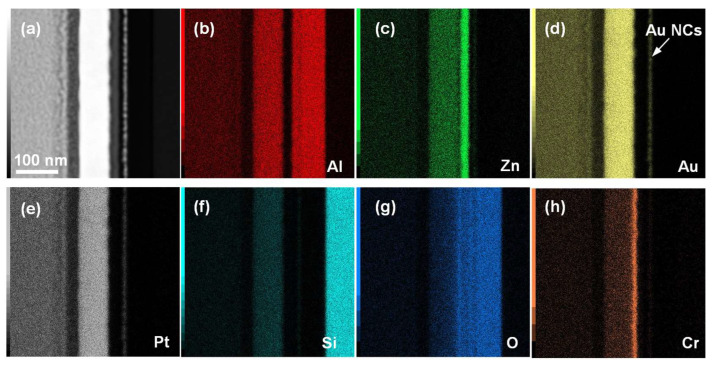
(**a**) Annular dark field image of the fabricated TFT memory; (**b**–**h**) EDS mapping profiles in the drain region, showing the distribution of Al, Zn, Au, Pt, Cr, Si, and O elements.

Figure 1b shows the SEM image of deposited 1.5 nm Au on the AlO_x_ blocking oxide layer after annealing for 1 min at 250 °C in a nitrogen atmosphere, together with the SEM image of the unannealed sample. It is evident that the deposited metal transformed from irregularly shaped islands to metal nanocrystals (NCs) with a round shape and uniform distribution after annealing. The average diameter of the fabricated Au NCs was 8 nm, with an areal density of 4.2 × 10^11^ cm^−2^. The formation of Au NCs can be interpreted as a self-assembly process, where the thermal energy given by annealing increases the surface mobility of the Au atoms, making them self-assemble into spherical nanocrystals with a lower total energy state [21]. Our results indicate that the process can occur even at a low temperature of 250 °C, which significantly reduces the thermal budget during device preparation and facilitates the integration of the memory devices on flexible substrates that cannot tolerate high temperatures. Au-O has a Gibbs free energy of −221.8 ± 20.9 kJ/mol, which is higher than that of Al-O with −511 ± 3 kJ/mol [22]. This facilitates the stabilization of Au NCs on AlO_x_, and the large difference in bond energy promotes the self-assembly of Au at relatively low temperature. The cross-sectional TEM image of the fabricated memory device was shown in Figure 1c, and the HR-TEM image of Au NCs in the device was shown in Figure 1d. A clear layered structure was observed, with Au NCs sandwiched between the amorphous AlO_x_ tunneling oxide layer and blocking oxide layer, which served as the material for charge storage. The HRTEM image of ZnO was shown in Supplementary Figure S2. It can be seen that the deposited ZnO had a polycrystalline morphology consisting of many columnar grains with different orientations. It was also observed that the ZnO channel was conformal and exhibited a distinct interface with the AlO_x_ tunneling layer, which facilitated the steep switching of the TFT memory under the gate control. Furthermore, Figure 1d reveals that the prepared Au NCs have a regular circular cross-section, which, in combination with the top-view SEM image in Figure 1b, suggests that the fabricated Au NCs were uniformly distributed, well-isolated, three-dimensional spheres. The annular dark field image of the fabricated TFT memory was illustrated in Figure 2a, and the EDS mapping profile of Al, Zn, Au, Cr, Si, and O elements are shown in Figure 2b–h. It can be seen that the TFT memory has a clear layered structure, and Au NCs were uniformly arranged in a consistent plane, isolated to an active channel by the AlO_x_ tunneling layer. The EDS spectrum for element analysis was given in Supplementary Figure S3.

**Figure 3:**
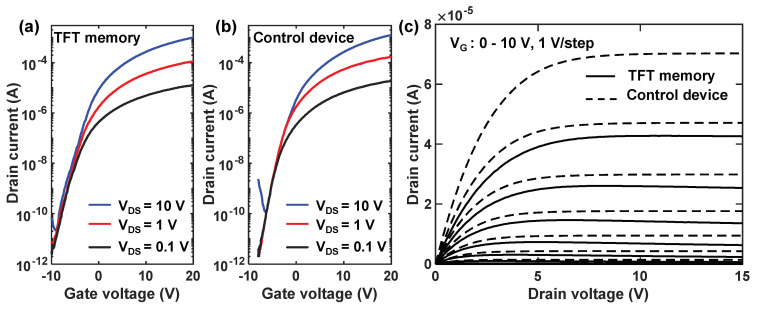
(**a**,**b**) Transfer curves of fabricated TFT memory and control device; (**c**) output curves of fabricated TFT memory and control device.

Figure 3a,b illustrates the transfer curves of TFT memory devices and control TFT devices at the initial state under different V_DS_. It can be seen that both devices exhibited good switching characteristics under the control of gate voltage, with the current on–off ratios both exceeding 10^7^. The extracted threshold voltage (V_th_) and subthreshold swing were −6.0 V, 0.71 V/dec for the TFT memory, and −4.8 V, 0.62 V/dec for the control device at V_DS_ = 1.0 V. Here, the threshold voltage is defined using the constant current method, which is the gate voltage when the drain-source current is equal to W/L × 10^−9^ A. The threshold of the memory device is slightly lower than that of the control device due to the fact that Au has a higher work function than the Fermi level of ZnO, which introduces an additional accumulation of electrons at the AlO_x_-ZnO interface. The field-effect mobility was obtained in the linear region by the following Equation (1) [34]:

(1) $$\mu_{\mathrm{FE}}=\frac{L}{{\mathrm{WC}}_{i}V_{\mathrm{DS}}}\cdot \frac{{\mathrm{dI}}_{D}}{{\mathrm{dV}}_{\mathrm{GS}}}\;$$

where the measured capacitance density of gate-stack C_i_ was 1.31 × 10^−7^ F/cm^2^ for the TFT memory and 1.47 × 10^−7^ F/cm^2^ for the control device. The extracted mobility was 17.6 cm^2^V^−1^s^−1^ for the TFT memory and 25.2 cm^2^V^−1^s^−1^ for the control device. Some degradation was observed in both the subthreshold swing and field-effect mobility of the TFT memory compared to the control device. One reason for this is that the presence of Au NCs increases the roughness of the AlO_x_-ZnO interface, leading to an increase in interface states at the AlO_x_-ZnO interface and a reduction in electron mobility within the ZnO channel. The above results show that the fabricated TFT memory exhibits superior open-state performance compared to those reported in the literature. Table 1 compares the on-state and memory performance of various oxide semiconductor TFT-based memories. The continuous deposition of AlO_x_ and ZnO by ALD without vacuum breaking ensures a sharp and smooth interface, with a large grain size for reducing the carrier scattering by grain border traps, ensuring fast transport of electrons in the accumulated channel. A suitable carrier density of 2.1 × 10^17^ cm^−3^ under the deposition temperature of 100 °C brings the memory good switching behavior with a high current on–off ratio. The benefits from using a non-destructive and low-temperature ALD technique show that improvements are significant for practical applications, as higher cell current enlarges the sensing margin of the readout circuit, facilitating fast access of the cell state and allowing for increasing the number of memory cells in a NAND string.

**Figure 4:**
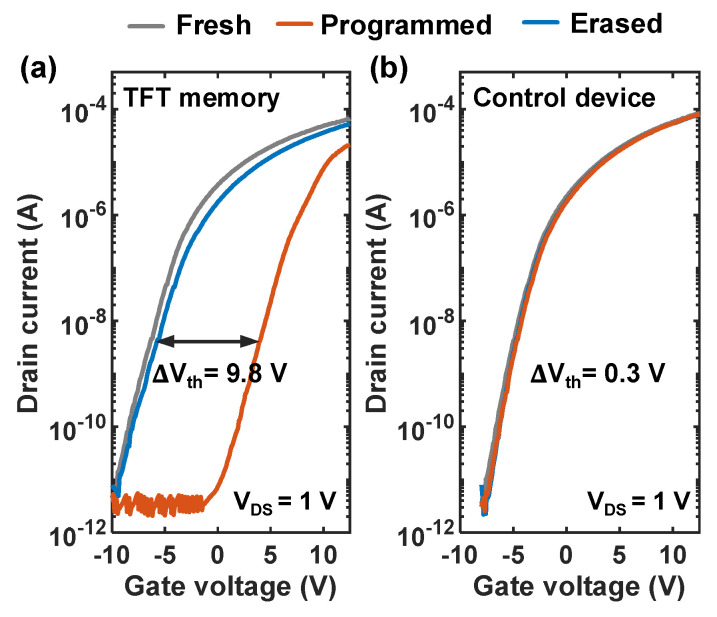
(**a**,**b**) Transfer curve of TFT memory and control device at fresh state, after 15 V, 100 ms programming and after −15 V, 1 s erasing.

Figure 4 shows the transfer curves of the TFT memory and control device at the initial state, programmed state, and erased state. The programming was performed by applying a voltage pulse of 15 V and 100 ms to the gate, while −15 V and 1 s were used for erasing, both with source and drain grounded. It can be seen that the V_th_ of the TFT memory shifted from −6.0 V to 4.0 V after programming and returned to −5.8 V after erasing. The P/E window of 9.8 V between the programmed state and erased state, compared to the 0.1 V P/E window of the control device, indicated the excellent memory performance of our TFT memory, which is attributed to the storage of electrons by Au NCs. Notably, no significant degradation in on-state performance was observed for both devices after programming and erasing, while the V_th_ of both devices was slightly higher than that in the fresh state after a series of erasing operations. One possible reason is that some electrons were trapped by some deep-level traps in the tunneling oxide during programming and cannot return to the channel after erasing. To evaluate the capability of Au NCs to trap electrons, the number of electrons trapped by NCs was calculated by Equation (2):

(2) $$n=\frac{C_{\mathrm{BO}}∆V_{\mathrm{th}}}{{q\sigma }_{\mathrm{NCs}}}$$

where C_BO_ is the capacitance density of the AlO_x_ blocking oxide, $∆V_{\mathrm{th}}$ is the P/E window, and $\sigma_{\mathrm{NCs}}$ is the density of Au NCs. According to the calculation, about 20 electrons were trapped by an Au NC after programming of 15 V, 0.1 s, which is higher than those reported in the literature [20,28,29]. Due to the high density of states around the Fermi level and stable chemical properties so that its surface properties will not be affected by annealing and subsequent processes, along with the appropriate average size of 8 nm and regular spherical morphology, our results show that the self-assembled Au NCs can provide large charge-trapping density, which ensures excellent non-volatility of the TFT memory. Furthermore, the power consumption during programming and erasing were calculated as 2.18 nJ and 2.13 nJ, respectively. Details of the calculation can be found in the Supplementary Information. If the power consumption needs to be further reduced, feasible approaches include reducing the P/E window by adjusting the P/E time, reducing P/E voltage, and shrinking the device size.

**Figure 5:**
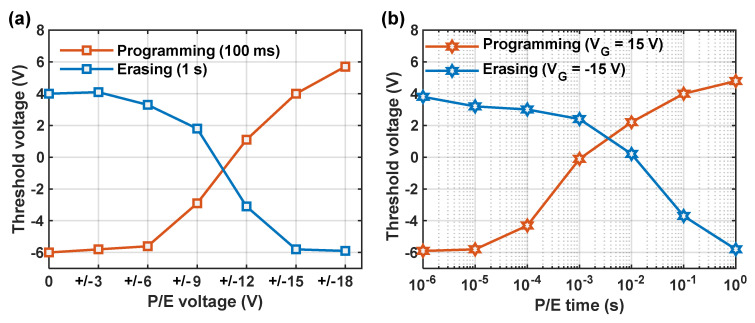
P/E characteristics of TFT memory. (**a**) V_th_ versus P/E voltage; (**b**) V_th_ versus P/E time.

Figure 5a illustrates the V_th_ of the memory cell under two conditions: fresh cell after a fixed programming time of 100 ms and programmed cell after erasure for 1 s. Various gate voltage amplitudes were applied for programming and erasing with the source and drain grounded. It can be observed that a significant shift in threshold voltage occurred for the fresh cell when the applied gate voltage exceeded 9 V, while for the erased state, V_th_ decreased with increasing erase voltage amplitude, and the shift became saturated at −18 V. Figure 5b shows the threshold voltage of the fresh cell and programmed cell of the TFT memory under 15V/−15V programming/erasing voltage amplitudes with different pulse widths. It is observed that the V_th_ shift of the fresh cell significantly increased with longer programming times beyond 0.1 ms, reaching 10 V at 100 ms. For the programmed cell, a significant V_th_ shift was observed after an erasing time exceeding 1 ms and reaching 9.8 V after erasing for 1 s. The above results show that the TFT memory has good programming and erasing performance. A large part of the reason for this, in addition to the high charge-trapping density of Au mentioned above, is attributed to the use of AlO_x_, a high-k dielectric with a measured k-value of 8.2, which significantly enhances the coupling between the control gate and the channel, resulting in obviously V_th_ shifts under gate voltage pulse.

**Figure 6:**
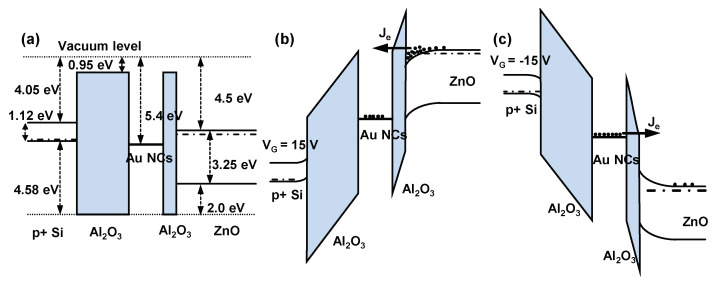
Band diagram of proposed ZnO TFT memory. (**a**) flatband; (**b**) programming; (**c**) erasing.

The programming and erasing mechanisms of the TFT memory can be elucidated through the band diagram of the device structure. Figure 6a–c illustrates the band diagram of the TFT memory structure at flat band, programming, and erasing. The band gap of ZnO is determined to be 3.25 eV by optical transmittance spectrum; details can be found in Supplementary Figure S4. As shown in Figure 6b, the Fowler–Nordheim (FN) tunneling was used for the programming of the device since a positive program voltage was applied to the gate while keeping the source and drain grounded. The FN tunneling current is notably influenced by the field strength of the tunneling oxide, which correlates with the applied write voltage [35]. As the programming voltage exceeded 9 V, the bending of the energy band allowed electrons to tunnel across the triangular barrier into Au NCs through FN tunneling, resulting in a significant change in V_th_. When erasing was performed, the negative gate voltage raised the energy level of Au NCs through capacitive coupling, and the charge stored in the NCs also increased the energy level of the NCs. Despite Au having a high work function of about 5.4 eV, the electrons stored in the Au nanocrystals can return to the channel through FN tunneling under a large gate voltage. This is evidenced by the observed strong electric field dependence of the threshold voltage shifts during erasing.

Furthermore, we observe that the programming efficiency of the TFT memory was higher than that of erasing, as programming always achieves the same V_th_ shift in less time. This is due to the asymmetric energy band structure of the device, as shown in Figure 6b,c, where the barrier between ZnO and AlO_x_ is 3.55 eV, while the barrier between Au and AlO_x_ is about 4.35 eV. Consequently, the FN tunneling current for programming is higher than that for erasing, resulting in a shorter programming time. Therefore, the programming and erasing time selected at a chosen P/E voltage of 15 V were 100 ms and 1 s, respectively, to ensure a stable P/E window during repetitive operations. In addition, our results indicate that although the hole barrier between ZnO and AlO_x_ is only 2 eV, erasing cannot be realized by hole injection because the observed V_th_ of erased cells is always higher than that of the fresh cells under various erasing settings. This difficulty arises from the inherent properties of ZnO as a wide-band semiconductor, which naturally exhibits strong n-type conducting, making it challenging to generate sufficient holes through inversion [36].

**Figure 7:**
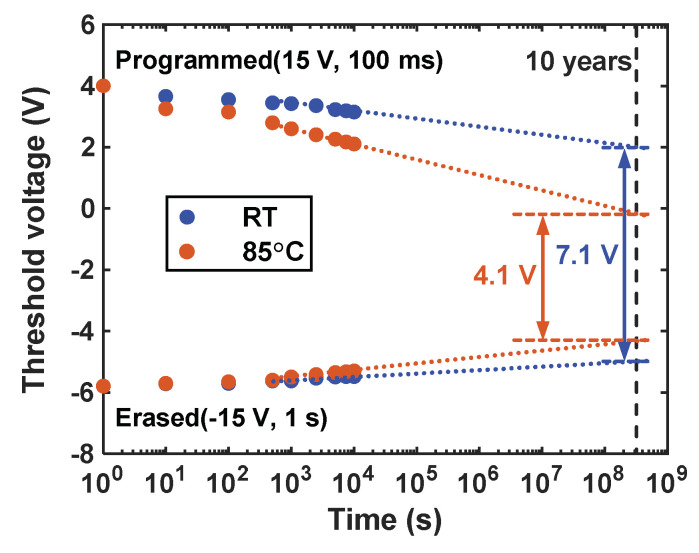
Retention characteristics of fabricated ZnO TFT memory under RT and 85 °C.

Figure 7 illustrates the retention characteristics of the TFT memory cell at room temperature and 85 °C. The V_th_ of the programmed cell and erased cell were monitored over time. At room temperature, it can be seen that the P/E window gradually decreased from 9.8 V to 8.6 V at 10^4^ s and further to 7 V when extrapolated to 10 years, representing a 29% loss of stored charge. At an elevated temperature of 85 °C, the P/E window decreased at a faster rate, with the extrapolated P/E window at 10 years reduced to 4 V, corresponding to a 59% loss of stored charge. The acceleration of charge loss with increasing temperature indicates a significant impact of temperature on the leakage current from the Au NCs to the channel under retention mode. Our prepared Au NCs TFT memory exhibits excellent retention properties for two main reasons. Firstly, the high work function of Au NCs enables the forming of a deep quantum well in AlO_x_, reducing the probability of electron tunneling. Secondly, the dense, pinhole-free, and insulated nature of the ALD-prepared AlO_x_ significantly reduces the leakage current from Au NCs, which is proven by the AFM (Multimode 8, Bruker, Billerica, MA, USA) image and current density curve of deposited AlO_x_ tunneling oxide in Supplementary Figure S5. 

**Figure 8:**
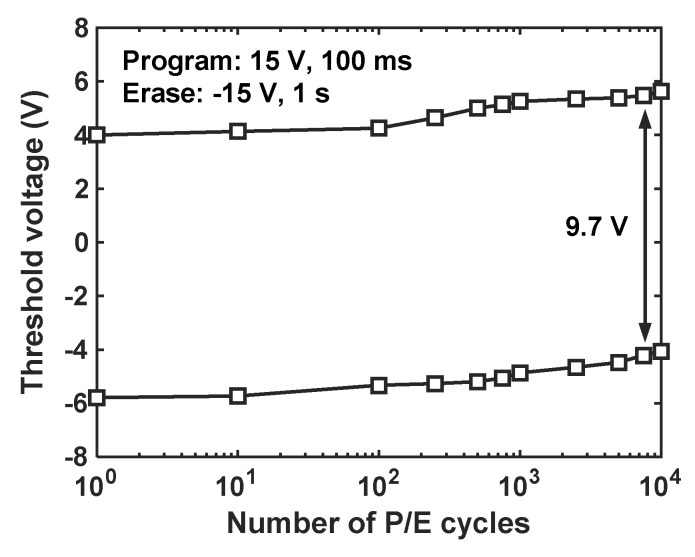
Endurance characteristics of fabricated ZnO TFT NCs memory.

Figure 8 shows the endurance characteristics of TFT memory. It can be observed that the V_th_ of both erased and programmed cells increased with the increase of P/E cycles, with the rate of change being larger after 500 cycles. Despite this, the TFT memory maintained a P/E window of 9.7 V after 10^4^ cycles. The shifts of V_th_ with the P/E cycle in both states were related to the degradation of tunneling oxide. Voltage stress in the P/E cycle induces trap generation in the tunneling oxide, which could capture electrons and lead to an increase in V_th_ of the memory over cycling. Moreover, unbalanced programming and erasing can also shift the V_th_ of erased cells and programmed cells in the same direction. To mitigate this, the voltage and time of programming and erasing should be more finely tuned. Overall, the high-quality AlO_x_ tunneling oxide enables the TFT memory to obtain robust endurance, making it favorable for practical applications.

## 4. Conclusions

In conclusion, we successfully fabricated a high-performance ZnO TFT non-volatile memory, with self-assembled Au NCs sandwiched between AlO_x_ and a maximum process temperature of 250 °C. The high work function and proven exceptional charge-trapping capability of the Au NCs provide the excellent non-volatility to the memory. The use of high-k dielectric AlO_x_ enhances the program and erase performance of the memory and ensures the long retention. Together with the low-temperature ALD ZnO channel, our TFT memory exhibits both excellent on-state performance and memory characteristics, including field-effect mobility, subthreshold swing, and current on–off ratio of 17.6 cm^2^V^−1^s^−1^, 0.71 V/dec, and 10^7^, respectively, and the P/E window of 9.8 V under 15 V, 100 ms programming and −15 V, 1 s erasing, which maintained to 7 V when extrapolated to 10 years. The memory demonstrated impressive endurance performance with a 9.7 V P/E window after 10^4^ P/E cycles. Combined with the low-temperature and large-scale fabrication capability of the process technology, our ZnO TFT memory exhibits great potential for integration into SoP and flexible electronic systems as a crucial component of memory modules.

## Figures and Tables

**Table 1:** Comparison of TFT non-volatile memories based on an oxide semiconductor channel.

Ref.	Gate Stacks	Channel	P/E Condition	∆V_th_	Charge Retention @10 yrs.	Mobility (cm^2^V^−1^s^−1^)	I_ON_/I_OFF_	SS(V/dec)	Max. Process Temp.
This work	AlO_x_/Au-NCs/AlO_x_	ZnO (ALD)	P: +15 V, 100 ms E: −15 V, 1 s	9.8 V	71 %	17.6	~10^7^	0.71	250 °C
[13]	Al_2_O_3_/Ni-NCs/Al_2_O_3_	a-IGZO (Sputtering)	P: +18 V, 5 ms E: −20 V, UV light, 100 s	13.8 V	52%	7.1	~10^7^	0.70	300 °C
[17]	HfLaO/M-OH	a-IGZO (Sputtering)	P: 10 V, 1 s E: −10 V, 1 s	1.5 V	78.9 %	3.9	2.7 × 10^5^	0.23	400 °C
[28]	HfO_2_/Pd-NCs/HfO_2_	ZnO (Sputtering)	P: +18 V, 200 ms E: −18 V, 200 ms	5.8 V	83%	N/A	~10^3^	1.9	RT

## Data Availability

The data are available on the request from the corresponding authors.

## Supplementary Materials

The following supporting information can be downloaded at: https://www.mdpi.com/article/10.3390/nano14080678/s1.
