# Supplementary material for: Non-Volatile Memory Based on ZnO Thin-Film Transistor with Self-Assembled Au Nanocrystals
Source: Nanomaterials (Basel). 2024 Apr 14;14(8):678. doi: 10.3390/nano14080678 (PMC11053943; doi:10.3390/nano14080678)
Supplement: Supplementary file 1 [file nanomaterials-14-00678-s001.zip › nanomaterials-2946525-supplementary.pdf]

Article

Non-volatile memory based on ZnO thin-film transistor with self-assembled Au nanocrystals

Hui Xie [1], Hao Wu [1,2,*] and Chang Liu [1]

[1] Key Laboratory of Artificial Micro- and Nano-structures of Ministry of Education, and School of Physics and Technology, Wuhan University, Wuhan 430072, China; h.sieh@whu.edu.cn (H.X.); chang.liu@whu.edu.cn (C.L.)
[2] Hubei Key Laboratory of Nuclear Solid Physics, Wuhan University, Wuhan 430072
* Correspondence: h.wu@whu.edu.cn (H.W.)

Supplementary Materials

Figure S1. Process flow of the proposed Au NCs ZnO TFT non-volatile memory device

Figure S2. HRTEM image of deposited ZnO film by ALD at 100℃

Figure S3. EDS spectrum from the EDS mapping of the device for element analysis

Figure S4. (a) Transmittance spectrum curve of fabricated ZnO on glass; (b) Tauc plot to determine the optical band gap of the fabricated ZnO

Figure S5. (a) AFM image of fabricated AlOx tunneling oxide; (b) Current density versus electrical field curves of the Au-Cr/AlOx/p++ Si structure, with 3 different measurement positions, which shows the uniform and consistent property

Table S1. ALD growth conditions of AlO_x and ZnO

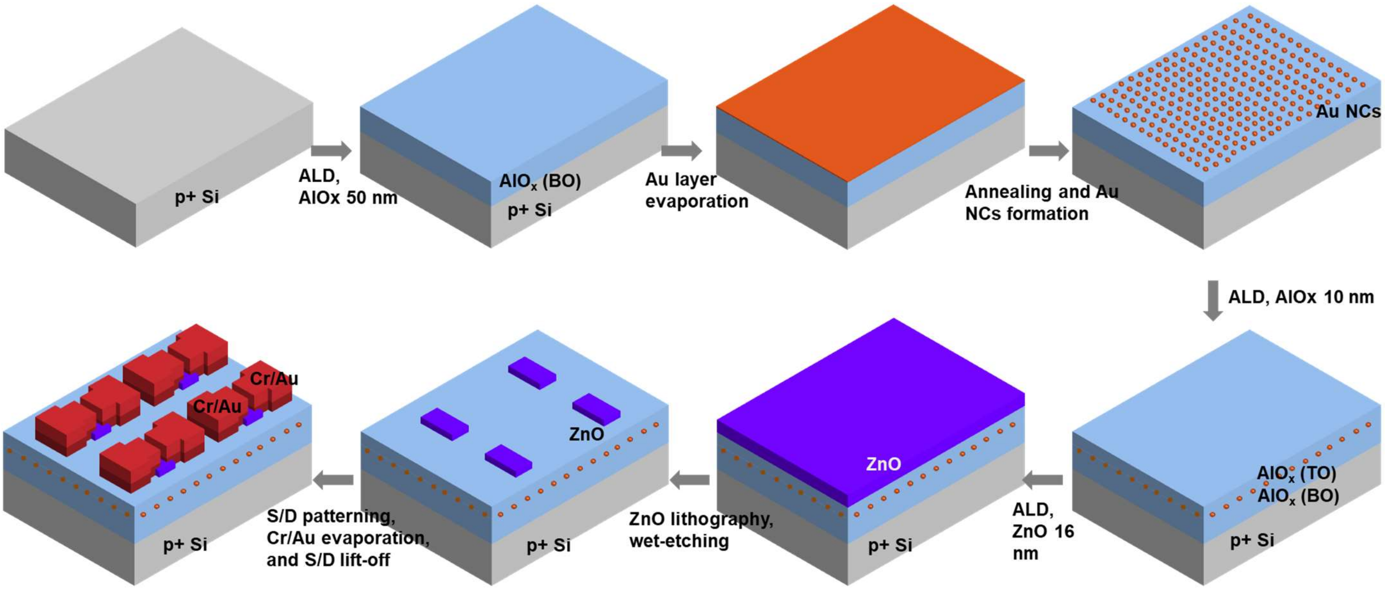

Figure S1. Process flow of the proposed Au NCs ZnO TFT non-volatile memory device

Table S1. ALD growth conditions of AlO_x and ZnO.

Material	Precursor	Exposure time	Purge time	Grow temperature	Chamber gas flow
AlOx	TMA	0.7 s	10 s	200℃	
	H_2O	1 s	15 s		
ZnO	DEZn	0.3 s	20 s	100℃	200 sccm
	H_2O	0.3 s	25 s		

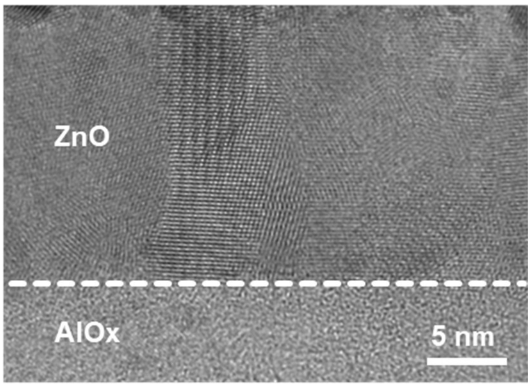

Figure S2. HRTEM image of deposited ZnO film by ALD at 100℃.

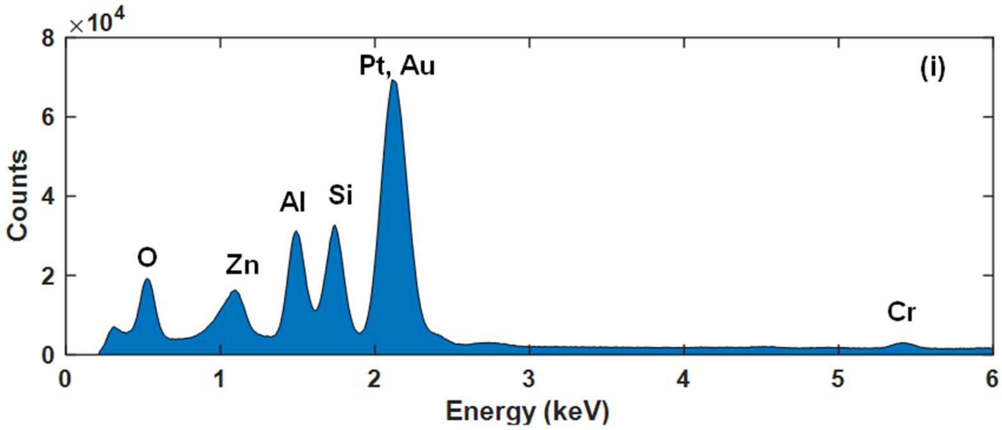

Figure S3. EDS spectrum from the EDS mapping of the TFT memory for element analysis

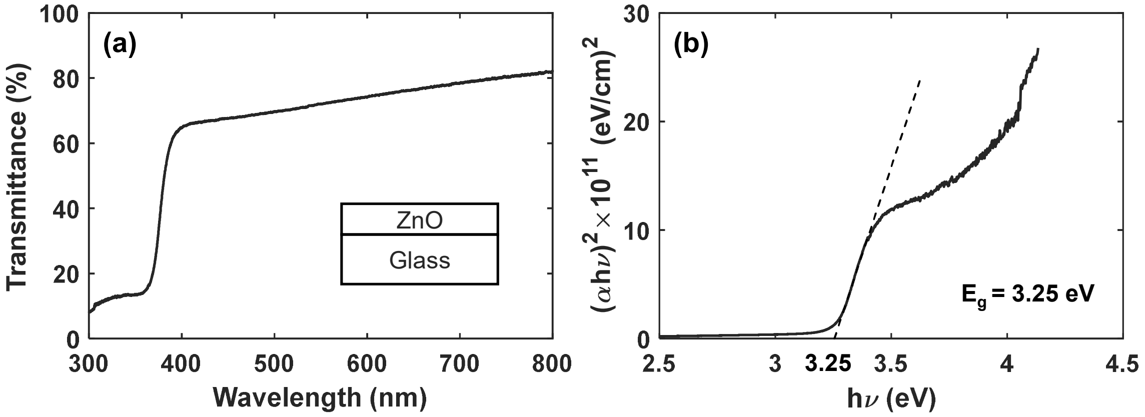

Figure S4. (**a**) Transmittance spectrum curve of fabricated ZnO on glass; (**b**) Tauc plot to determine the optical band gap of the fabricated ZnO

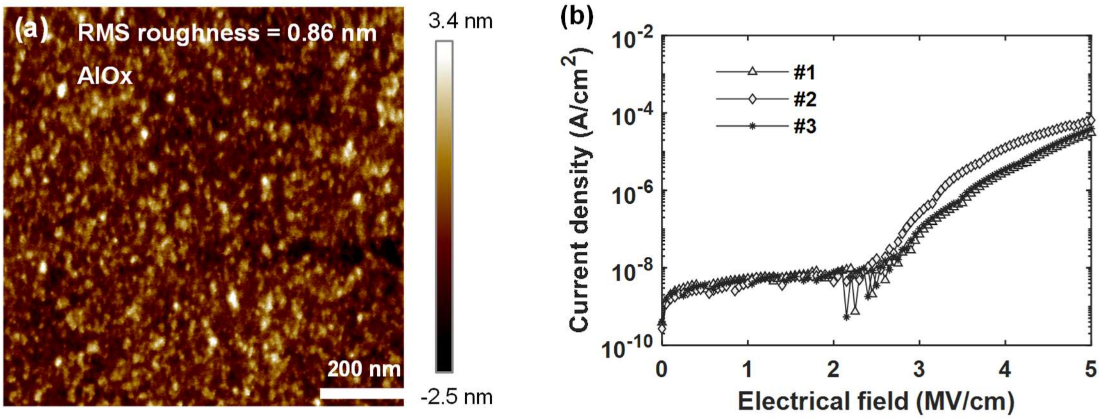

Figure S5. (**a**) AFM image of fabricated AlOx tunneling oxide; (**b**) Current density versus electrical field curves of the Au-Cr/AlOx/p++ Si structure, with 3 different measurement positions, which shows the uniform and consistent property

Calculation procedure of the power consumption of the TFT memory during programming and erasing

During programming and erasing, the power consumption can be calculated by

$$W_P = V_P \int_0^{t_P} I_P(t)dt = V_P Q_e = V_p q N_{e,P}$$

$$W_E = V_E \int_0^{t_E} I_E(t)dt = V_E Q_E = |V_E| q N_{e,E}$$

V_P = 15 V, the program voltage

V_E = -15 V, the erase voltage

$N_{e,P}$: number of electrons trapped by Au NCs under 15 V/100 ms programming

$N_{e,E}$: number of electrons detrapped from Au NCs under -15 V/1 s erasing

$$N_{e,P} = C_{BO} \cdot \frac{\Delta V_{th,P}}{q}$$

$$N_{e,E} = C_{BO} \cdot \frac{\Delta V_{th,E}}{q}$$

Where

$C_{BO} = 1.45\times10^{-11}$ F, measured capacitance of 50 nm AlO_x blocking oxide

$\Delta V_{th,P}$ = 10 V, threshold voltage shift from fresh state to programmed state under 15 V/100 ms programming

$\Delta V_{th,\ E}$ = 9.8 V, threshold voltage shift from programmed state to erases state under -15 V/1 s programming

Thus

$$W_P = V_P C_{BO} \Delta V_{th,P} = 15\ \mathrm{V} \times 1.45 \times 10^{-11}\ \mathrm{F} \times 10\ \mathrm{V} = 2.18\ \mathrm{nJ}$$

$$W_E = |V_E| C_{BO} \Delta V_{th,E} = 15\ \mathrm{V} \times 1.45 \times 10^{-11}\ \mathrm{F} \times 9.8\ \mathrm{V} = 2.13\ \mathrm{nJ}$$
